# Impact of GLO1 Knock Down on GLUT4 Trafficking and Glucose Uptake in L6 Myoblasts

**DOI:** 10.1371/journal.pone.0065195

**Published:** 2013-05-23

**Authors:** Britta Engelbrecht, Bernd Stratmann, Cornelius Hess, Diethelm Tschoepe, Thomas Gawlowski

**Affiliations:** 1 Ruhr-University Bochum, Diabetes Center, Heart and Diabetes Center NRW, Bad Oeynhausen, Germany; 2 Institute of Forensic Medicine, University of Bonn, Bonn, Germany; Universitat de Barcelona, Spain

## Abstract

Methylglyoxal (MG), a highly reactive α-dicarbonyl metabolite of glucose degradation pathways, protein and fatty acid metabolism, plays an important role in the pathogenesis of diabetic complications. Hyperglycemia triggers enhanced production of MG and increased generation of advanced glycation endproducts (AGEs). In non-enzymatic reactions, MG reacts with arginine residues of proteins to form the AGEs argpyrimidine and hydroimidazolone. Glyoxalase 1 (GLO1), in combination with glyoxalase 2 and the co-factor glutathione constitute the glyoxalase system, which is responsible for the detoxification of MG. A GLO1 specific knock down results in accumulation of MG in targeted cells. The aim of this study was to investigate the effect of intracellularly accumulated MG on insulin signaling and on the translocation of the glucose transporter 4 (GLUT4). Therefore, L6 cells stably expressing a *myc*-tagged GLUT4 were examined. For the intracellular accumulation of MG, GLO1, the first enzyme of the glyoxalase pathway, was down regulated by siRNA knock down and cells were cultivated under hyperglycemic conditions (25 mM glucose) for 48 h. Here we show that GLO1 knock down augmented GLUT4 level on the cell surface of L6 myoblasts at least in part through reduction of GLUT4 internalization, resulting in increased glucose uptake. However, intracellular accumulation of MG had no effect on GLUT4 concentration or modification. The antioxidant and MG scavenger NAC prevented the MG-induced GLUT4 translocation. Tiron, which is also a well-known antioxidant, had no impact on MG-induced GLUT4 translocation.

## Introduction

Diabetes mellitus is characterized by chronic hyperglycemia which is associated with excessive formation of advanced glycation endproducts (AGEs) and increased plasma levels of the highly reactive α-dicarbonyl methylglyoxal (MG) [1], [2], [3]. Formation of AGEs is involved in the pathogenesis of several diabetic complications, concerning the microvascular as well as the macrovascular system comprising coronary artery disease, cerebral and peripheral artery disease, neuropathy, nephropathy and cataracts [4], [5], [6], [7]. MG is a toxic by-product of several metabolic pathways, mainly from glycolysis, but also from lipid peroxidation and threonine catabolism [8], [9], [10], [11]. MG is probably the most reactive intracellular AGE precursor. It reacts readily with and thereby modifies certain proteins, lipids, and DNA and alters their normal structure and/or function [9], [12]. Different studies have shown that MG levels are significantly increased in plasma and tissues of diabetic patients, which leads to increased AGE accumulation. Their presence has been linked to the pathogenesis of diabetic complications [1], [2], [3], [13]. Formation of protein derived AGEs causes structural distortion, loss of side chain charges and functional impairments, such as enzyme inactivation [14], [15], [16]. Naturally, MG is mainly detoxified by the glutathione dependent glyoxalase pathway, involving glyoxalase 1 (GLO1) and glyoxalase 2 to prevent accumulation of MG in mammalian cells [17], [18], [19], [20]. Glycation of proteins by MG and other dicarbonyls may increase reactive oxygen species (ROS), such as superoxide, hydrogen peroxide and hydroxyl radicals, and thereby may be source or contributory factor of oxidative stress. ROS are able to modulate a number of cell signaling events leading to activation or inhibition of kinases and phosphatases, redox sensitive transcription factors and genes [3], [21]. Recent studies showed that in adipocytes and muscle cells induction of oxidative stress by incubation with H_2_O_2_ increases glucose uptake and stimulates GLUT4 translocation [22], [23], [24]. Insulin is involved in a variety of biological responses in regulatory pathways, including glucose metabolism, protein synthesis and cellular proliferation as well as differentiation [25], [26]. The main action of insulin is the regulation of cellular glucose uptake into skeletal muscle cells and adipocytes via glucose transporter 4 (GLUT4) translocation from intracellular vesicles to the plasma membrane [27], [28]. This dynamic process is retained through a continuous recycling and relocation of GLUT4 between the plasma membrane and intracellular compartments. [28], [29], [30], [31]. In a non-stimulated state only 2–5% of the GLUT4 content is presented on the cell surface. Insulin stimulation induces the release of GLUT4 via exocytosis, and cellular GLUT4 content becomes exposed at the cell surface [28], [30], [31]. In both cell types, glucose uptake is accomplished via activation of insulin receptor substrate 1 (IRS-1), the phosphatidylinositol-3-kinase (PI3-Kinase), and Akt. All components of the PI3-Kinase/Akt-mediated signal pathway take part in the GLUT4 translocation [30], [31], [32], [33]. Patients with diabetes mellitus present reduced insulin-induced GLUT4 translocation and glucose uptake resulting in elevated plasma glucose concentrations. Impairment in insulin signaling events, such IRS-1 and Akt phosphorylation are detected [34]. Yet, the mechanisms of insulin resistance remain poorly understood; both, genetic and environmental factors are being discussed in this clinical setting [35], [36]. Recent studies point to the close relationship of MG accumulation with insulin resistance and lead to the assumption that MG might have inhibitory effects towards insulin signaling [37], [38], [39].

To further analyze the effects of MG on insulin signaling we investigated the impact of intracellular accumulation of MG by GLO1 knock down on glucose uptake and GLUT4 translocation in the rat skeletal muscle cell line L6-GLUT4*myc*, that stably expresses a *myc*-tagged GLUT4. Our results show that increased GLUT4 translocation to the plasma membrane was detected after GLO1 knock down which was accompanied by increased glucose uptake, independently of MG-induced oxidative stress or modification of GLUT4. However, the GLO1 knock down had no impact on IRS-1 expression and phosphorylation. In conclusion, the GLO1 knock down results in impaired internalization of GLUT4, which results in higher GLUT4 density in the cell membrane.

## Materials and Methods

### Cell culture

L6-GLUT4*myc*-tagged myoblasts (kindly provided by Prof. Amira Klip, University of Toronto, Canada) were used as a cell culture model. L6-GLUT4*myc* cells were maintained in α-minimal essential medium (α-MEM, Invitrogen, Carlsbad, USA) supplemented with 10% (v/v) fetal bovine serum (FBS, PAA Laboratories, Cölbe, Germany) and antimycotic/antibiotic solution (100 units/mL penicillin, 100 µg/mL streptomycin, 250 ng/mL amphotericin B, Invitrogen, Carlsbad, USA) at 37°C in a humidified atmosphere of 5% CO_2_. Cells were grown to 70–80% confluence.

### Gene specific knock-down using siRNA

For transient transfection L6-GLUT4*myc* cells were grown in 6-well plates at a density of 1×10^5^ cells per well. Cells were transfected with 75 nM siRNA (final concentration, Ambion, Austin, USA) directed against GLO1 or with a non-silencing scrambled siRNA as control, using Lipofectamine™ 2000 transfection reagent (Invitrogen, Carlsbad, USA) according to the manufacturer's instructions. 4 µL of siRNA (50 µM) and 3 µL of transfection reagent Lipofectamine™ 2000 were diluted with 247 µL Opti-MEM, and incubated for 20 min at room temperature, and then added to the cells. 1.5 mL transfection-medium (α-MEM with 10% FBS but without antibiotic) were added to each culture well. 24 h after transfection, the medium was replaced by fresh complete medium with 25 mM glucose. Assays were done 72 h after transfection. For insulin stimulation, transfected cells were incubated in serum-free α-MEM for 4 h prior to stimulation with 100 nM insulin for 1 h.

### Immunoblot analysis

Cells were washed twice with ice-cold PBS and lysed using lysis buffer containing sodium chloride (150 mM), Tris-HCl (50 mM), NaF (1 mM), EDTA (1 mM), Na_3_VO_4_ (1 mM), Nonidet P40 (1%), sodium deoxycholate (0,25%) and proteinase inhibitor cocktail (Sigma-Aldrich, Saint Louis, USA), repeatedly frozen and finally sonicated. The total protein concentrations were determined by bicinchoninic acid assay (Sigma-Aldrich, St. Louis, USA) using BSA for a standard curve. Proteins were separated by NuPAGE® 4–12% Bis-Tris gel (Invitrogen, Carlsbad, USA) electrophoresis and electrotransferred (Trans-blot SD, Biorad, Hercules, USA) to nitrocellulose membranes (Biorad, Hercules, USA). Membranes were incubated for 1 h in blocking-solution containing 5% dry milk in Tris-buffered saline. Membranes were washed in Tris-buffered saline and incubated overnight at 4°C with the following antibodies: monoclonal anti-Akt1 (1∶5000; R&D Systems, Minniapolis, USA), monoclonal anti-Akt2 (1∶1000; Cell Signaling Tech, Massachusetts, USA) polyclonal anti-phospho-totalAkt (S473) (1∶1000; Abcam, Cambrige, UK), polyclonal anti-IRS1 (1∶1000; Cell Signaling Tech, Massachusetts, USA) and polyclonal anti-phospho-IRS1 (Y612) (1∶1000; Abcam, Cambrige, UK). After washing, the membranes were incubated for 1 h with horseradish peroxidase (HRP)-conjugated secondary antibodies. As a HRP substrate Chemiglow (Alpha Innotech Corporation, San Leandro, USA) was used and recorded by a CCD-camera system (Fluor Chem FC3, CellBiosciences, Santa Clara, USA).

### Detection of oxidative stress by flow cytometry

Intracellular ROS formation was evaluated using the peroxide-dependent oxidation of 2′-7′-dichlorofluorescein-diacetate (DCFH-DA). DCFH-DA is cleaved intracellularly by nonspecific esterases to form DCFH, which is further oxidized by ROS to form the fluorescent compound DCF. This assay is used in numerous publications, even in the direct stimulation of cells with AGE-BSA [40]. Characteristic excitation and emission wavelengths of AGE fluorescence are 360–370 nm for excitation and 430–450 nm for emission, respectively. Thus, there is no overlap of spectra with DCF fluorescence which spectra characteristics are 480 nm for excitation and 530 nm for emission. SiRNA-transfected cells were washed twice with Hank's buffered saline solution (HBSS, pH 7.4 Invitrogen, Carlsbad, USA). Afterwards, 2′-,7′-dichlorodihydrofluorescein diacetate (DCF-DA, Sigma-Aldrich, St. Louis, USA) was added followed by incubation at 37°C for 30 min in the dark. Cells were then collected after trypsin incubation, suspended in HBSS buffer, centrifuged (5 min; 400× *g*) and suspended again in 0.5 mL HBSS buffer. Fluorescence intensity of 5,000 cells was measured by flow cytometry (FACScan, Becton Dickinson, Franklin Lakes, USA), comparable to the work of Lee et al. [40].

### Detection of apoptosis by flow cytometry

SiRNA-transfected cells were double labeled with annexin V–FITC and propidium iodide (PI) (Beckman Coulter, Krefeld, Germany), according to the manufacturer's instructions, and immediately analyzed by flow cytometry (FACScan, Becton Dickinson, Franklin Lakes, USA). The amount of apoptotic cells was calculated as the percentage of the number of annexin V positive cells over the total number of the cells.

### Detection of surface GLUT4*myc* by flow cytometry

Cell surface GLUT4*myc* was quantified using antibody-coupled flow cytometry assays. Following transfection with siRNA and incubation for 4 h in serum-free α-MEM at 37°C, cells were collected after trypsin incubation and suspended in serum-free α-MEM containing 100 nM insulin and incubated for 1 h at 37°C. The cells were fixed with 1% paraformaldehyde for 30 min at 37°C, blocked with 1% BSA before reacting with anti-c-*myc*(FITC) antibody (1∶100 in 1% BSA, Sigma-Aldrich, St. Louis, USA) for 1 h at 37°C. Cells were washed two times with PBS and suspended again in 0.5 mL PBS. Fluorescence intensity of 5,000 cells was measured by flow cytometry.

### Glucose uptake measurement

The glucose uptake was measured with the fluorescent deoxyglucose analog 2-NBDG (2-(N-(7-nitrobenz-2-oxa-1,3-diazol-4-yl)amino)-2-deoxyglucose, Invitrogen, Carlsbad, USA) by flow cytometry. SiRNA transfected cells were incubated with 50 µM 2-NBDG in α-MEM (5 mM glucose) for 1 h. The 2-NBDG uptake reaction was stopped by washing the cells twice with pre-cooled PBS. Cells were collected after incubation with trypsin, suspended in PBS buffer, centrifuged (5 min; 400× *g*) and suspended again in 0.1 mL PBS buffer. Propidium iodide (PI) staining was used to exclude necrotic cells. Fluorescence intensity of 5,000 cells was measured by flow cytometry.

### Measurement of GLUT4*myc* internalization

GLUT4*myc* internalization was measured using antibody-coupled flow cytometry assays. Following siRNA knock down and incubation for 4 h in serum-free α-MEM at 37°C, cells were collected after trypsin incubation and suspended in serum-free α-MEM and were incubated 30 min at 37°C. Cells were rinsed with ice-cold PBS and incubated with the anti-c-*myc*(FITC) antibody (1∶100 in 1% BSA) for 1 h at 4°C. The cells were washed two times with ice-cold PBS and the surface-labeled GLUT4*myc* was allowed to internalize by re-warming the cells to 37°C in pre-warmed serum-free α-MEM. At indicated time points, cells were placed on ice, washed with ice-cold PBS, fixed with 2% paraformaldehyde for 15 min and washed two times with PBS and suspended again in 0.5 mL PBS. Fluorescence intensity of 5,000 cells was measured by flow cytometry.

### Chromatographic and mass spectrometric measurement of intracellularly accumulated MG

The detection of intracellularly accumulated MG was done by HPLC-MS as described elsewhere [41]. Briefly, siRNA-transfected cells were lysed in 1 mL using lysis buffer containing imidazol (10 mM), sucrose (300 mM), NaF (10 mM) and EDTA (1 mM) and immediately processed. 100 µL supernatant was diluted with 400 µL water and spiked with internal standard (1 µg/mL 3,4-hexandione in methanol). After precipitation MG was liberated from its high protein binding by adding 250 µL of perchloric acid (7%). The sample was then mixed for 10 s, left for 15 min and centrifuged for 10 min at 10,000 g. Derivatization took place with 100 µL of 2,3-diaminonaphthalene (1 mg/mL) overnight at 4°C after adjustment of pH to 6.5 with saturated sodium hydrogencarbonate solution. After extraction with 4 mL ethyl acetate the organic layer was evaporated and reconstituted in 200 µL of methanol. The samples were than separated chromatographically and molecules were ionized by electrospray ionization (ESI) in positive mode.

Studies were made on a Shimadzu (Duisburg, Germany) LC 20 series (binary pump, degasser, controller and autosampler) coupled with an Applied Biosystems API 4000 QTrap Mass Spectrometer (Applied Biosystems, Darmstadt, Germany). Chromatographic separation was carried out with a Phenomenex (Aschaffenburg, Germany) Synergi® MAX-RP analytical column (150×2 mm, 4 µm particle size) and a Phenomenex C_12_ (4×2 mm) guard column. A gradient flow (0.4 mL/min) of 5 mM ammoniumformiate in water pH 3.5 (eluent A) to 5 mM ammoniumformiate in methanol (eluent B) was applied as follows: 70% eluent B to 90% eluent B in 7 min, 90% eluent B to 70% B in 0.5 min, 2.5 min equilibrating time at 70% eluent B. Molecules were ionized by electrospray ionization (ESI) in positive mode. Mass spectrometry parameters were optimized by infusing a solution of 2,3-diaminonaphthalene derivatized MG or internal standard (IS) directly into the ion source. Data was acquired using Analyst Software 1.5.1 (Applied Biosystems, Darmstadt, Germany). Calibration curves were generated using the peak area ratio of the target peak of the MG derivative to IS derivative target peak and were fitted with a linear regression. The limit of detection of this procedure is 1.3 ng/ml, limit of quantification 3.2 ng/ml. The linearity ranges from 5 ng/ml–500 ng/ml, and the precision data at low (15 ng/ml), middle (60 ng/ml) and high (125 ng/ml) concentration are as follows: intraday imprecision: 10.3%, 9.2% and 8.3%, interday imprecision: 15.3%, 14.2% and 9.4%, and accuracy bias: −5.7%, −3.0% and 7.4%.

### GLUT4 immunoprecipitation

L6 cells were transfected with siRNA as described above. *Myc*-tagged GLUT4 (GLUT4*myc*) stably expressed in L6 cells was immunoprecipitated via the *myc* epitope by the anti-c-*myc* Immunoprecipitation Kit (Sigma-Aldrich, St. Louis, USA), according to the manufacturer's instructions. Immunoprecipitation of GLUT4 was performed with 150 µg of the whole protein.

Immunoprecipitated protein was denatured in Laemmli sample buffer and resolved by SDS/PAGE and electrotransferred (Trans-blot SD, Biorad, Hercules, USA) to nitrocellulose membranes as described above. Membranes were incubated overnight at 4°C with the polyclonal anti-GLUT4 (1∶20, Chemicon Merck Millipore, Bellerica, USA) and mouse monoclonal anti-MG-H1 IgG (1∶2,000, kindly provided by Prof. Michael Brownlee, New York, USA).

### Statistics

Results of the experimental studies are reported as mean ± SEM. Differences between discrete treatments were analyzed by Student's t-Test or one-way ANOVA followed by Dunnet's multiple comparison post-test using GraphPad Prism version 5.02 for Windows (GraphPad Software, San Diego, USA). P values <0.05 were regarded statistically significant. In the following N describes independent biological experiments, whereas n accounts for the number of repetitions of measurements.

## Results

The impact of the intracellular accumulation of MG in L6 myoblasts by specific siRNA knock down of GLO1 on GLUT4 translocation, modification and glucose uptake as well as IRS-1 and Akt expression and phosphorylation as molecular events in insulin signaling was analyzed.

### GLUT4 translocation and 2-NBDG uptake

The translocation of GLUT4 in L6 GLUT4*myc* myoblasts from cytoplasm to the cell membrane was determined by flow cytometry using an anti-c-*myc*(FITC) monoclonal antibody (see Figure S1).

**Figure 1:**
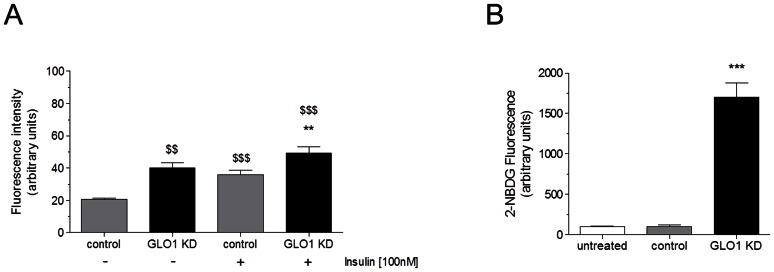
GLUT4 translocation and 2-NBDG uptake. (A) Change of fluorescence intensity anti-c-*myc*(FITC) stained in L6 cells after specific siRNA knock down of GLO1 with and without insulin stimulation for 1 h. (N = 5, n = 3). ^**^p<0.01 vs. control siRNA with insulin. ^$$^p<0.01 and ^$$$^p<0.001 vs. control siRNA. Means ± SEM. (B) L6 cells staining with 2-NBDG for 1 h after GLO1 knock down (following 72 h siRNA transfection, and 48 h high glucose cultivation). Fluorescence intensity was measured by flow cytometry. ^***^p<0.001 vs. control siRNA. (N = 3, n = 3). Means ± SEM.

To examine the impact of GLO1 knock down on GLUT4 translocation the cells were transfected with 75 nM siRNA directed against GLO1 or with a non-silencing scrambled siRNA as control. We tested three different GLO1 siRNA constructs, which all resulted in more than 80% down-regulation of GLO1 on mRNA and protein level (see Figure S2). As shown in Figure 1A siRNA mediated knock down of GLO1 resulted in an enhanced GLUT4 translocation in non-stimulated as well as in insulin stimulated state. Following GLO1 knock down the level of GLUT4*myc* translocation was significantly increased compared to cells transfected with scrambled siRNA (40.1 ± 3.3 vs. 20.6 ± 0.6, p<0.01). In cells with GLO1 knock down, insulin stimulation further increased the translocation of GLUT4 (49.3 ± 3.9 a.u., p<0.001). Next, we examined if the increased translocation of GLUT4 is mirrored by enhanced glucose uptake in L6 myoblasts. The glucose uptake was analyzed by measuring the uptake of the fluorescent glucose analogue 2-NBDG. 2-NBDG staining of GLO1 knock down cells (following 72 h siRNA transfection and 48 h high glucose cultivation) showed a significant increase in 2-NBDG uptake without insulin stimulation, compared to scrambled siRNA transfected cells under the same cultivation conditions (100.0 ± 24.6 vs. 1,701.3 ± 175.8 a.u., p<0.001) (Figure 1B).

### Influence of GLO1 knock down on insulin signaling in L6 myoblasts: Expression and phosphorylation of IRS-1, Akt1, and Akt2

**Figure 2:**
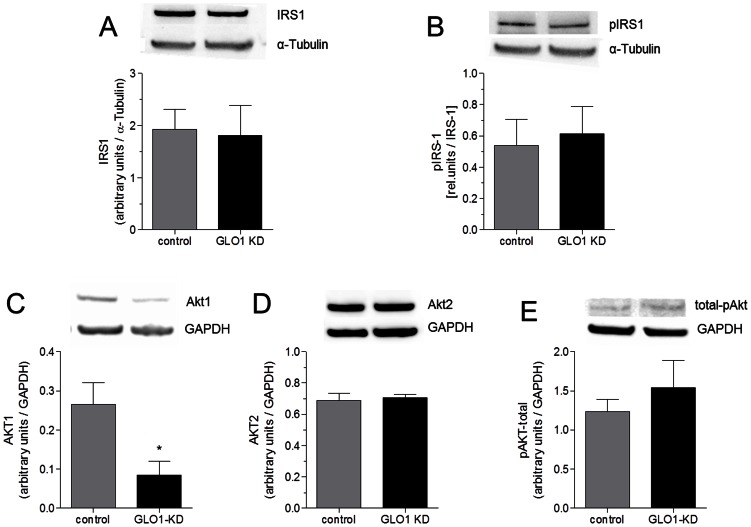
Expression and phosphorylation of IRS-1, Akt1 and Akt2 as well as phosphorylation totalAkt. (A) Representative Western Blot analysis of IRS-1 expression in L6 cells after intracellular accumulation of MG by specific siRNA knock down of GLO1 (following 72 h siRNA transfection and 48 h high glucose cultivation). (N = 5). Means ± SEM. (B) Representative Western Blot analysis of pIRS-1 expression in L6 cells after intracellular accumulation of MG by specific siRNA knock down of GLO1 (following 72 h siRNA transfection and 48 h high glucose cultivation). (N = 5). Means ± SEM. (C) Representative Western Blot analysis of Akt1 expression in L6 cells after intracellular accumulation of MG by specific siRNA knock down of GLO1 (following 72 h siRNA transfection and 48 h high glucose cultivation). ^*^p<0.05 vs. control siRNA. (N = 6). Means ± SEM. (D) Representative Western Blot analysis of Akt2 expression in L6 cells after intracellular accumulation of MG by specific siRNA knock down of GLO1 (following 72 h siRNA transfection and 48 h high glucose cultivation). (N = 6). Means ± SEM. (E) Representative Western Blot analysis of total-pAkt expression in L6 cells after intracellular accumulation of MG by specific siRNA knock down of GLO1 (following 72 h siRNA transfection and 48 h high glucose cultivation). (N = 4). Means ± SEM.

Translocation of GLUT4 to the plasma membrane is regulated via activation of IRS-1, PI3-kinase, and Akt. To examine if MG-induced GLUT4 translocation is mediated by enhanced activation of the insulin receptor or downstream processes in the insulin signaling cascade we determined the expression levels of IRS-1, Akt1 and Akt2 as well as the phosphorylation status of IRS-1 and totalAkt. GLO1 knock down did not impair expression of IRS-1, the first downstream signaling member of the insulin receptor and also had no effect on the phosphorylation of IRS-1 (Figure 2 A&B).

As Akt is considered to be the upstream regulator of GLUT4 translocation, we studied the effect of intracellular accumulation of MG via GLO1 knock down and high glucose cultivation conditions on Akt1 and Akt2 expression and totalAkt activation in response to insulin. The activation of Akt is regulated by phosphorylation of specific serine and threonine residues. To investigate the ability of MG to affect Akt signalling, Akt phosphorylation on Serin^473^ was measured. After 72 h transfection with siRNA and 48 h high glucose cultivation L6 cells presented a significant 3.75-fold decrease in Akt1 expression (0.08±0.02 a.u., p<0.05) compared to scrambled siRNA transfected cells (0.30±0.05 a.u.) (Figure 2C). The transfection of GLO1-specific siRNA did neither impair the expression of Akt2 nor the phosphorylation of totalAkt in L6 myoblasts (Figure 2D&E).

### GLO1 knock down induces oxidative stress in L6 myoblasts

**Figure 3:**
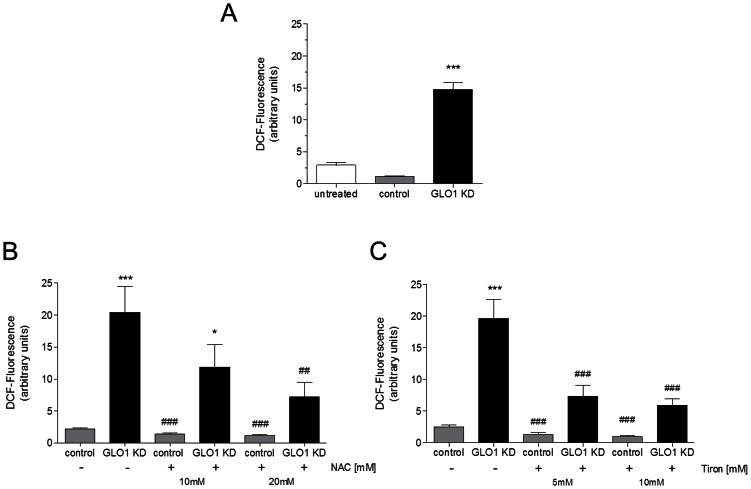
Flow cytometric ROS analyses of DCF stained L6 cells. (A) Mean fluorescence intensity of L6 cells with GLO1 knock down (following 72 h siRNA transfection, and 48 high glucose cultivation). ^***^p<0.001 vs. control. (N = 5, n = 3). Means ± SEM. Mean fluorescence intensity of L6 cells with GLO1 knock down (following 72 h siRNA transfection, and 48 high glucose cultivation) and incubation with (B) NAC (10 and 20 mM, 24 h) (N = 4, n = 3) or (C) tiron (5 and 10 mM, 24 h). (N = 4, n = 3).^*^p<0.05 and ^***^p<0.001 vs. control. ^##^p<0.01 and ^###^p<0.001 vs. GLO1 KD. Means ± SEM.

Several studies showed that oxidative stress impairs insulin-induced GLUT4 translocation. Next, we investigated whether oxidative stress could be responsible for the MG induced increases of GLUT4 translocation. Intracellular ROS formation in GLO1 siRNA treated L6 cells was evaluated using the peroxide-dependent oxidation of 2′-,7′-dichlorofluorescein-diacetate (DCFH-DA). The resulting fluorescent compound DCF was measured by flow cytometry. MG does not react directly with DCF-DA and thus do not falsify the results (not shown). Increased generation of ROS was detected in independently performed experiments (Figure 3A). NAC and tiron are well known potential antioxidants, which are also capable of inhibiting apoptosis. In addition to its antioxidant properties, NAC is also a well-recognized MG scavenger. Using both antioxidants, we were able to show that ROS production is prevented in GLO1 knock down L6 myoblasts.

Incubation with 10 mM NAC decreased oxidative stress in GLO1 knock down cells 1.7-fold (11.9±3.5 a.u.) and incubation with 20 mM NAC led to a 2.8-fold decrease (7.2±2.3 a.u., p<0.01) (compared to GLO1 knock down without NAC (Figure 3B). Co-incubation with 5 mM tiron resulted in significant decrease of oxidative stress if compared to GLO1 knock down without tiron (7.3±1.7 a.u. vs. 20.4±4.1 a.u., p<0.001). Incubation with 10 mM tiron decreased the oxidative stress 3.5-fold compared to L6 cells with GLO1 knock down but without tiron (5.9±1.0 a.u. vs. 20.4±4.1 a.u. p<0.001) in L6 myoblasts (Figure 3C).

### Oxidative stress is not responsible for the MG-induced GLUT4 translocation

Next, we examined if preventing of MG-induced ROS accumulation by co-incubation with NAC or tiron has an impact on the translocation of GLUT4.

**Figure 4:**
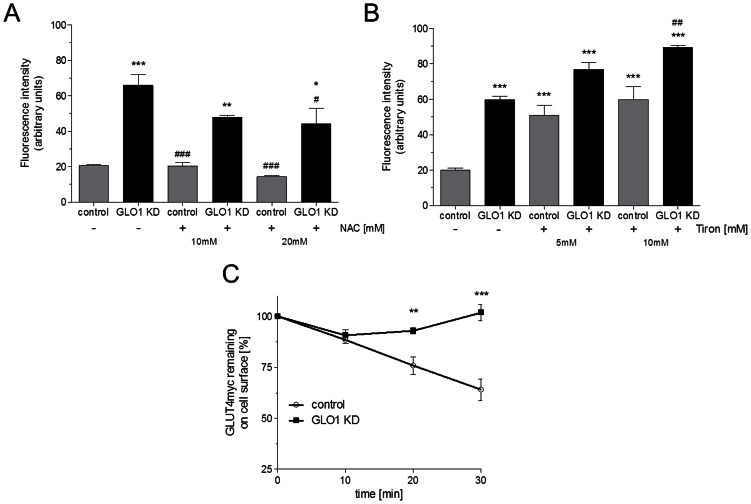
Effects of antioxidant substances on GLUT4 translocation and GLUT4 endocytosis. Mean fluorescence intensity of anti-c-*myc*(FITC) on L6 cells with GLO1 knock down (following 72 h siRNA transfection, and 48 h high glucose cultivation) incubated with (A) NAC (10 and 20 mM, 24 h) (N = 3, n = 2) or (B) tiron (5 and 10 mM, 24 h) (N = 3, n = 3). ^*^p<0.05, ^**^p<0.01 and ^***^p<0.001 vs. control. ^#^p<0.05, ^##^p<0.01 and ^###^p<0.001 vs. GLO1 knock down. Means ± SEM. (C) GLUT4*myc* endocytosis was measured by flow cytometry. Change of fluorescence intensity anti-c-*myc*(FITC) stained in L6 cells after intracellular accumulation of MG by specific siRNA knock down of GLO1. (N = 3, n = 3). ^**^p<0.01 and ^***^p<0.001 vs. control siRNA. Means ± SEM.

Incubation with the antioxidant NAC for 24 h prevents the GLUT4 translocation in siRNA transfected cells (10 mM NAC: 47.7 ± 1.2 a.u.; 20 mM NAC: 44.0 ± 8.8 a.u. vs. 65.9 ± 6.2 a.u., p<0.05 for each). However, incubation with the antioxidant tiron did not prevent the GLUT4 translocation in siRNA treated L6 myoblasts. Incubation with 10 mM tiron showed even a significant increase in GLUT4 translocation in high glucose cultivated cells and GLO1 knock down (GLO1 knock down + tiron vs. GLO1 knock down: 89.2 ± 1.1 a.u. vs. 597.0 ± 1.9 a.u., p<0.01 (Figure 4 A&B).

### GLO1 knock down impairs GLUT4 internalization

MG-induced elevation in surface GLUT4 may result from an increase in GLUT4 exocytosis, a reduction in GLUT4 internalization by endocytosis, or both. To determine GLUT4 recycling in GLO1 knock down L6 cells, we measured GLUT4*myc* internalization at different time points by anti-c-*myc*(FITC) monoclonal antibody in the flow cytometer. In untreated cells GLUT4*myc* was internalized rapidly. GLO1 knock down reduced on the GLUT4 internalization process and GLUT4 was largely retained at the cell surface. GLO1 knock down resulted in 101.8±4.0% (compared with 64.0±5.3% in scrambled siRNA transfected cells, p<0.001) presence of GLUT4 at the cell surface after 30 min (Figure 4C). GLO1 knock down resulted in complete loss of GLUT4 internalization after 30 min.

### Intracellular accumulation of MG by GLO1 knock down

**Figure 5:**
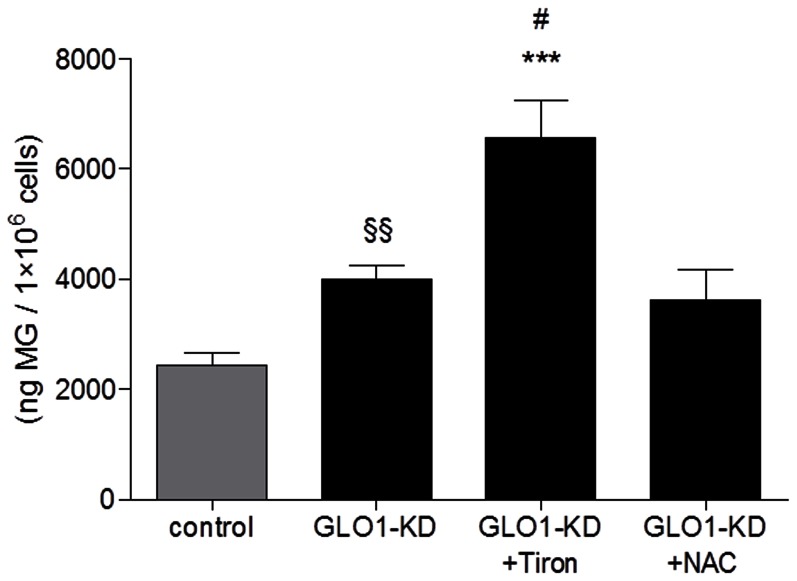
Measurement of intracellular MG. Intracellular MG accumulation following GLO1 knock down was measured by LC/MS. (N = 4).^***^p<0,001 vs. control by ANOVA. ^#^p<0.05 vs. GLO1 knock down by ANOVA. ^§§^p<0.01 vs. control (by t-test). Means ± SEM.

Knock down of GLO1 was effectively proven on RNA and protein level (see Figure S2) and resulted in a significant increase in MG concentration compared to control (p = 0.0082 by t-test vs. control) (Figure 5). Incubation for 24 h with the antioxidant tiron resulted in significant increase in MG levels in GLO1 siRNA transfected L6 myoblasts (p<0.001 vs. control, p<0.05 vs. GLO1 knock down by ANOVA). NAC incubation resulted in a slight, but non-significant reduction in MG level compared to GLO1 knock down. In addition, we could show that the intracellular accumulation of MG by knock down of GLO1 did neither result in an increase of GLUT4 concentration nor in pronounced MG-H1 modification (data not shown).

### GLO1 knock down induced apoptosis in L6 myoblasts

**Figure 6:**
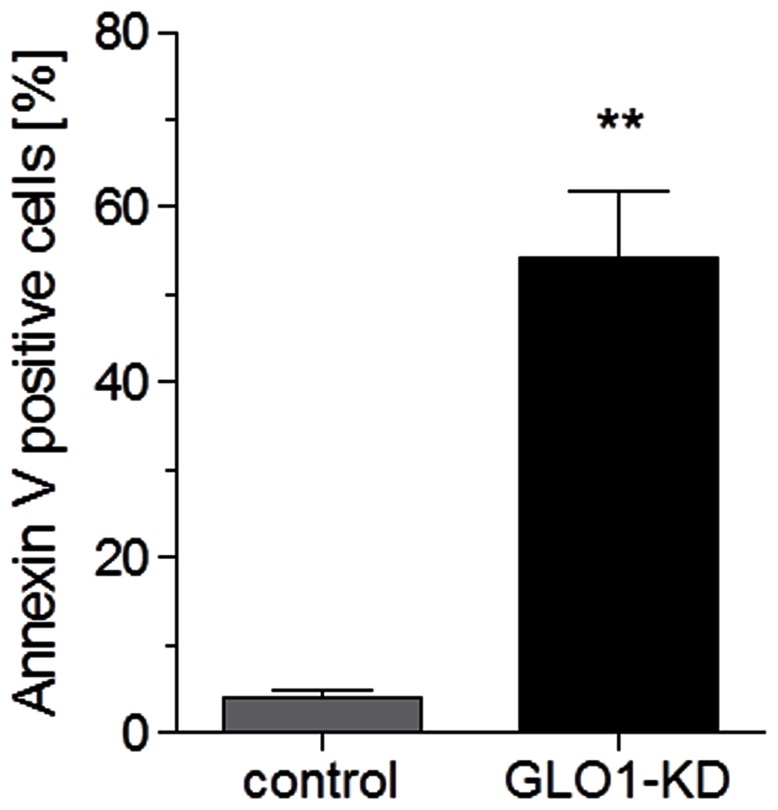
Induction of apoptosis by MG. Ratio of apoptotic cells with GLO1 knock down (following 72 h siRNA transfection, and 48 high glucose cultivation) (N = 3, n = 3). ^**^p<0.01 vs. control. Means ± SEM.

As Akt1, an anti-apoptotic protein was down regulated by GLO1 knock down investigations of apoptosis by flow cytometry were performed and clearly demonstrated that intracellular accumulation of MG by GLO1 knock down resulted in an significant increase in apoptosis (13.3 fold, respectively) (Figure 6).

## Discussion

As a complex metabolic disorder diabetes mellitus is characterized by chronic hyperglycemia due to defects in insulin secretion or insulin resistance. Chronic hyperglycemia leads to increased intracellular accumulation of MG, a reactive dicarbonyl which reacts with proteins, nucleic acids and lipids to form AGEs [42], [43], [44]. To clarify the correlation between hyperglycemia induced MG accumulation in cells and insulin resistance, we investigated the impact of impaired MG levels in GLUT4 translocation and glucose uptake. For this purpose specific siRNA knock down of GLO1 was induced in L6 myoblasts to provoke an intracellular accumulation of MG. MG accumulation by GLO1 knock down was proven by measurements of intracellular MG by HPLC/mass spectrometry. The GLO1 knock down resulted in increased GLUT4 level on the plasma membrane. Intracellularly accumulated MG did not lead to an increased GLUT4 expression or MG-H1 modification. Thus, our observations clearly rely on the disturbances in GLUT4 trafficking, and not on the modification of the protein.

In contrast to the investigations of Riboulet-Chavey et al. we could also detect a significant increase in glucose uptake [39]. Riboulet-Chavey et al. found that insulin signaling is inhibited in short-term MG-exposed L6 myoblasts, shown by decreased IRS-1 phosphorylation in MG-treated cells, resulting in impaired glucose uptake. Our results show that GLO1 knock down (72 h) have no effect on IRS-1 expression or phosphorylation, and increase GLUT4 translocation and glucose uptake.

To investigate if MG has an impact on PI3 kinase signaling pathway, we also determined the expression and activity of Akt in PI3-kinase signaling, which is an important modulator of insulin signaling, cell proliferation and survival. Insulin signaling pathways are implicated in a wide range of cellular mechanisms controlling cell metabolism, growth, and differentiation. The serine/threonine kinase Akt is activated by insulin and insulin-like growth factors as a downstream target of PI3-Kinase [45], [46].

In eukaryote tissue, three isoforms of Akt exist of which only Akt1 and Akt2 are ubiquitously expressed in all tissue types examined so far [46], [47]. Several studies have demonstrated that Akt2 is important for insulin induced translocation of GLUT4 [48], [49], [50]. Immunoblotting analysis of Akt1 and Akt2 were performed to investigate the expression of both isoforms. There was neither impact of MG on activation of totalAkt by Serin^473^ (Ser^473^) phosphorylation nor on the expression of Akt2 but expression of Akt1 was significantly decreased in L6-cells with GLO1 knock down. Akt1 is involved in cellular survival pathways. The analysis by flow cytometry clearly demonstrated an increase of apoptosis in GLO1 knock down cells. The decreased expression of Akt1 upon MG accumulation may thus contribute to increased apoptosis in L6 myoblasts.

Several studies have shown that ROS-induced oxidative stress has been implicated in insulin signaling pathways and can induce glucose uptake and GLUT4 translocation [22], [23], [24], [51], [52]. Accumulation of MG in cells leads to increased production of ROS and oxidative stress [53], [54]. To clarify if the MG-induced increase in GLUT4 translocation and glucose uptake are results of enhanced oxidative stress, L6 cells were pre-treated with the antioxidants NAC or tiron in this investigation. Pre-treatment with both antioxidants prevents the MG-induced oxidative stress in GLO1-siRNA-treated L6 cells. However, only pre-treatment with NAC could prevent MG-induced GLUT4 translocation. The effect of NAC can be explained partly by the fact that NAC - in addition to its role as an antioxidant - is also known to be a MG scavenger [55]. NAC pre-treatment may thus prevent MG-induced GLUT4 translocation by scavenging intracellular MG. The detection of intracellular MG following GLO1 knock down in case of NAC incubation by our methods revealed no reduction in total MG levels. This may be due to the fact that the applied test measured total intracellular MG, protein bound as well as free MG, and thus cannot distinguish between free and NAC-bound MG. Assuming that NAC binds intracellular MG, the amount of free MG is reduced if compared to GLO1 knock down and can explain the beneficial effect of NAC in this experimental setting.

Pre-incubation with tiron did not prevent MG-induced GLUT4 translocation. Tiron, as an antioxidant but not a MG scavenger, could prevent the oxidative stress but has no effect on MG-induced GLUT4 translocation. Surprisingly, pre-treatment with tiron resulted in a more pronounced MG production following GLO1-knock down, which may at least in part explain the fact that tiron pre-treated cells present more GLUT4 on the cell surface than cells without pre-treatment with tiron. These results show that the MG-induced GLUT4 translocation is not mediated by the oxidative stress.

A major goal of this study was to map the effect of MG on the mechanisms of GLUT4 traffic. The glucose transporter GLUT4 relocates continuously between the cell surface and intracellular stores. This cycling consists of exocytotic movements of GLUT4 within endosomal vesicles towards the plasma membrane, and endocytotic movements back to the intracellular stores. Defects in this cycling are associated with insulin resistance [28], [56]. To determine if MG has an impact of GLUT4 cycling, we measured GLUT4*myc* endocytosis in GLO1-siRNA-treated L6 myoblasts to untreated cells. The results revealed that GLO1 knock down impairs the internalization of GLUT4, which results in prolonged presentation of GLUT4 at the cell surface. Therefore, we hypothesize that the MG related effect; the gain in GLUT4 on the cell surface is achieved primarily by reducing the rate of GLUT4 endocytosis. By which mechanisms MG exactly affects the endocytosis of GLUT4 has to be elucidated in more detail in future studies. A recently published study showed that short-term incubation with very high MG concentration (10 mM) increases GLUT4 and GLUT1 endocytosis [57]. It seems like acute and chronic high MG level have the opposite impact on the glucose uptake into the cell. A possible reason for different impact of acute or chronic high MG level could be the MG-derived formation of AGEs. MG reacts with proteins and modulates their normal structure and function. The level of MG-modifications is concentration and time-dependent. Chronically elevated MG level, found in diabetes, result in accumulation of MG-derived protein modification. It remains to be elucidated if MG-modification of proteins, which are involved in the GLUT4 cycling, could be responsible for the MG-induced inhibition of GLUT4 internalization.

However, our data clearly show that the knock down of GLO1, a MG-detoxifying enzyme, affects the GLUT4 cycling, resulting in increased GLUT4 level on the cell surface and increased glucose uptake. This effect does not depend on the modification of GLUT4 by MG, which only takes place at high amounts of MG (own observations). GLUT4 cycling is a fine-tuned process and disruption of this process can have pathological consequences.

## Supporting Information

Figure S1 **Measurement of GLUT4 translocation with and without insulin stimulus.** (A) Change of fluorescence intensity in anti-c-*myc*(FITC) stained L6 cells with and without insulin stimulus. (N = 5, n = 2). ^**^p<0.01 vs. untreated. Means ± SEM. (B) Representative histograms of fluorescence changes in anti-c-*myc*(FITC) stained L6 cells with and without insulin stimulus. Black line: L6 cells without insulin stimulus, gray line: L6 cells with insulin stimulus.
(TIF)

Figure S2 **Analysis of GLO1 Expression.** (A) Representative western blot analysis performed with anti-GLO1 (Untr.  =  untreated cells, Ctr.  =  scrambled siRNA, A-C  =  three different GLO1 siRNAs) (B) Western Blot analysis for GLO1 of L6 cells transfected with three different GLO1 siRNA (following 72 h siRNA transfection). (N = 3). ^**^p<0.01 vs. control. Means ± SEM. (C) mRNA Expression of GLO1 in L6 cells transfected with three different GLO1 siRNA (following 72 h siRNA transfection). (N = 3, n = 2). ^***^p<0.001 vs. control. Means ± SEM.
(TIF)
