# Supplementary figures and images for: Impact of GLO1 Knock Down on GLUT4 Trafficking and Glucose Uptake in L6 Myoblasts
Source: PLoS One. 2013 May 23;8(5):e65195. doi: 10.1371/journal.pone.0065195 (PMC3662699; doi:10.1371/journal.pone.0065195)

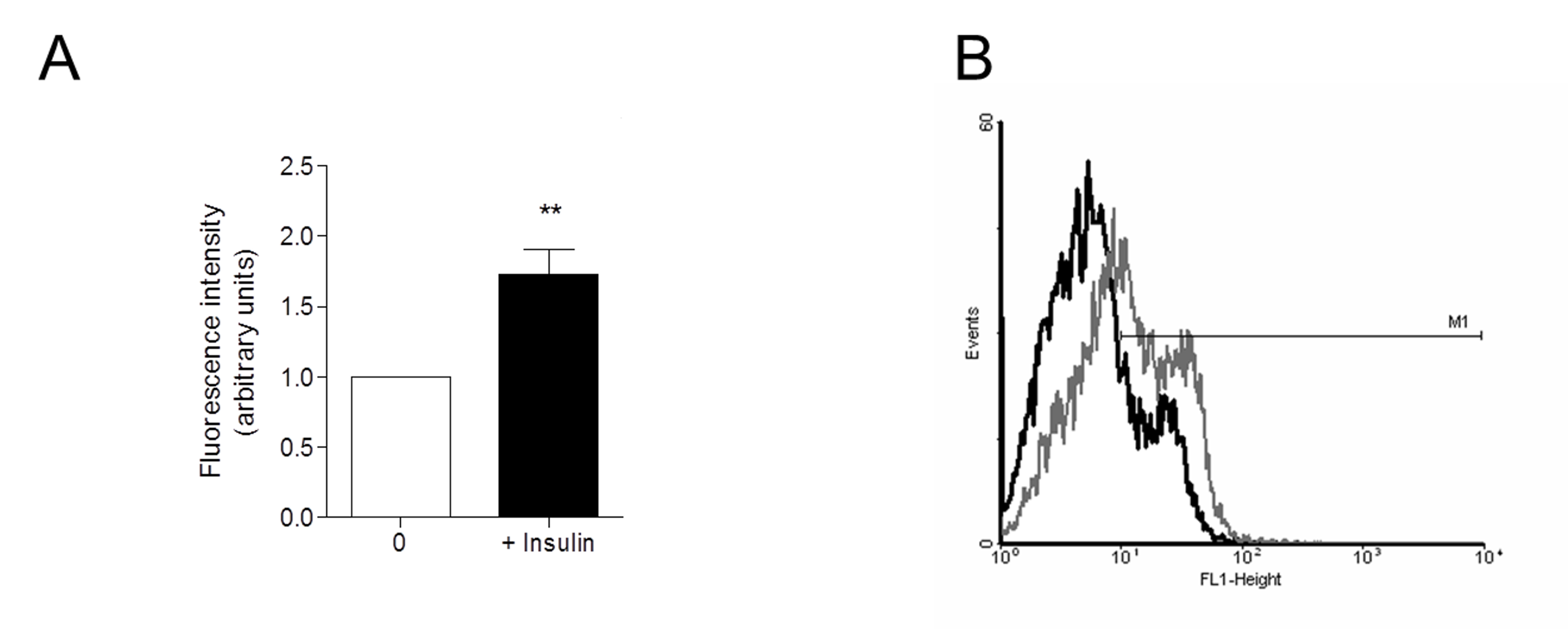

Supplement: Figure S1 — Measurement of GLUT4 translocation with and without insulin stimulus. (A) Change of fluorescence intensity in anti-c-myc(FITC) stained L6 cells with and without insulin stimulus. (N = 5, n = 2). **p<0.01 vs. untreated. Means ± SEM. (B) Representative histograms of fluorescence changes in anti-c-myc(FITC) stained L6 cells with and without insulin stimulus. Black line: L6 cells without insulin stimulus, gray line: L6 cells with insulin stimulus. (TIF) [file pone.0065195.s001.tif]
